# Prospective feasibility study on the efficacy and safety of a novel spiral dilator for endoscopic ultrasound‐guided drainage

**DOI:** 10.1002/deo2.170

**Published:** 2022-10-17

**Authors:** Takahisa Ogawa, Yoshihide Kanno, Shinsuke Koshita, Hiroaki Kusunose, Toshitaka Sakai, Keisuke Yonamine, Kazuaki Miyamoto, Fumisato Kozakai, Haruka Okano, Hideyuki Anan, Kento Hosokawa, Kei Ito

**Affiliations:** ^1^ Department of Gastroenterology Sendai City Medical Center Miyagi Japan

**Keywords:** endoscopic ultrasound‐guided biliary drainage, endoscopic ultrasound‐guided gallbladder drainage, endoscopic ultrasound‐guided hepaticogastrostomy, endoscopic ultrasound‐guided pancreatic duct drainage, Tornus ES

## Abstract

**Objectives:**

A difficult step in endoscopic ultrasound (EUS)‐guided drainage procedures is dilation of the puncture tract before stent deployment. The efficacy and safety of a novel spiral dilator, Tornus ES, for EUS‐guided drainage were investigated in this study.

**Methods:**

This study was conducted as a prospective, single‐arm, observational study at Sendai City Medical center. Dilation of the puncture tract using a spiral dilator was attempted for all EUS‐guided drainage cases. The primary outcome was the technical success rate which was defined as successful stent placement in the puncture tract. Secondary outcomes were the success rate of dilation using a spiral dilator, procedure time, and adverse events related to the procedures.

**Results:**

A total of 10 patients were enrolled between January and March 2022. Seven patients underwent EUS‐guided biliary drainage (hepaticogastrostomy for six and hepaticojejunostomy for one), and the remaining three patients underwent EUS‐guided gallbladder drainage. The technical success rate and the success rate of dilation using a spiral dilator were both 100%. The mean procedure time was 27 min. No adverse events related to the procedure occurred in all cases.

**Conclusions:**

Dilation of the puncture tract using a spiral dilator was effective and safe and might make it easier to perform EUS‐guided drainage.

## INTRODUCTION

Endoscopic ultrasound (EUS)‐guided biliary drainage (EUS‐BD), such as EUS‐guided hepaticogastrostomy (EUS‐HGS), EUS‐guided choledochoduodenostomy, and EUS‐guided hepaticojejunostomy (EUS‐HJS), has become widely used over the last decade as salvage therapy when transpapillary drainage is unsuccessful.^1^, ^2^, ^3^, ^4^ In addition, given the results of several studies, EUS‐BD has the potential of becoming the primary biliary drainage method.^5^, ^6^, ^7^ Furthermore, the number of studies on EUS‐guided drainage techniques, including gallbladder drainage (EUS‐GBD), pancreatic duct drainage (EUS‐PD), and pancreatic pseudocyst drainage (EUS‐PCD), have been reported at an accelerating rate over the last decade.^8^, ^9^, ^10^

The process of puncture tract dilation is one of the hardest steps making EUS‐guided drainage technically difficult.^11^, ^12^, ^13^, ^14^, ^15^, ^16^ To resolve this issue, a novel spiral dilator for EUS‐guided drainage has recently been developed.^15^, ^16^ The aim of this prospective study was to evaluate the efficacy and safety of this new dilator for EUS‐guided drainage.

## METHODS

### Study design

This feasibility study was a prospective, single‐arm study conducted at Sendai City Medical Center between January and March 2022. The study was approved by the institutional review board of Sendai City Medical Center (approval number, 2022‐0003) and registered in the University Hospital Medical Information Network (UMIN) clinical trial registry (UMIN000046410).

### Patients

Consecutive patients who met the following inclusion criteria and who did not meet any of the exclusion criteria were enrolled in this study. Inclusion criteria were: (1) patients who would undergo any EUS‐guided drainage, including EUS‐BD, EUS‐GBD, EUS‐PD, and EUS‐PCD, and (2) those who gave written informed consent about participating in the study. Exclusion criteria were as follows: (1) bleeding tendency, (2) poor general condition (the Eastern Cooperative Oncology Group performance status 3 or 4),^17^ (3) massive ascites around the planned puncture site, and (4) pregnancy. Patients who had previously undergone transpapillary or percutaneous drainage were not excluded from the study. The sample size of this first feasibility study was determined to be 10 after discussion among the participating doctors.

### Spiral dilator evaluated

**FIGURE 1:**
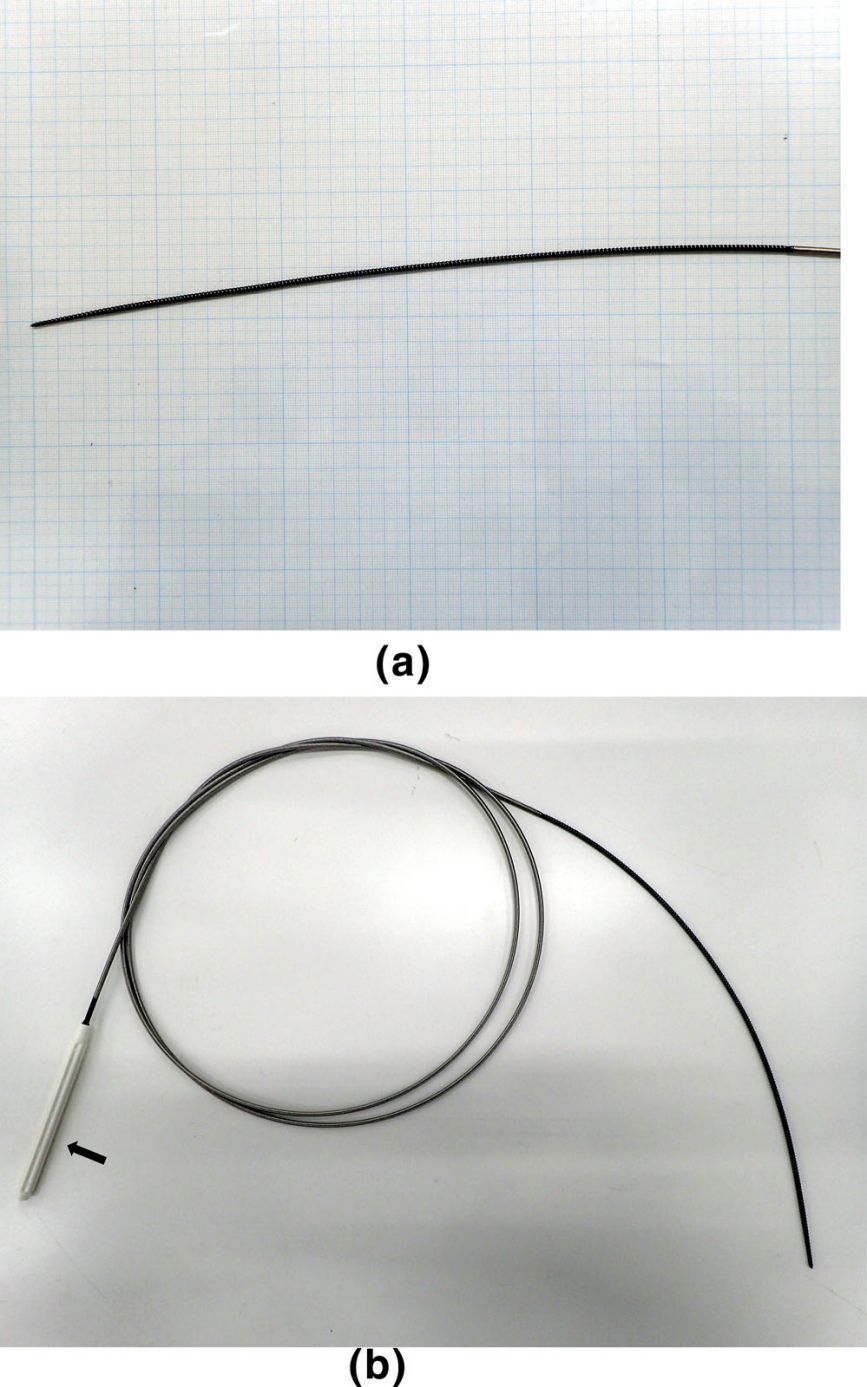
Novel spiral dilator Tornus ES (Olympus Co., Tokyo, Japan). (a) The distal part of this dilator is screw‐shaped. The outer diameter is 7Fr, and the tip is tapered. (b) The dilator is advanced by turning the handle (arrow) clockwise

**FIGURE 2:**
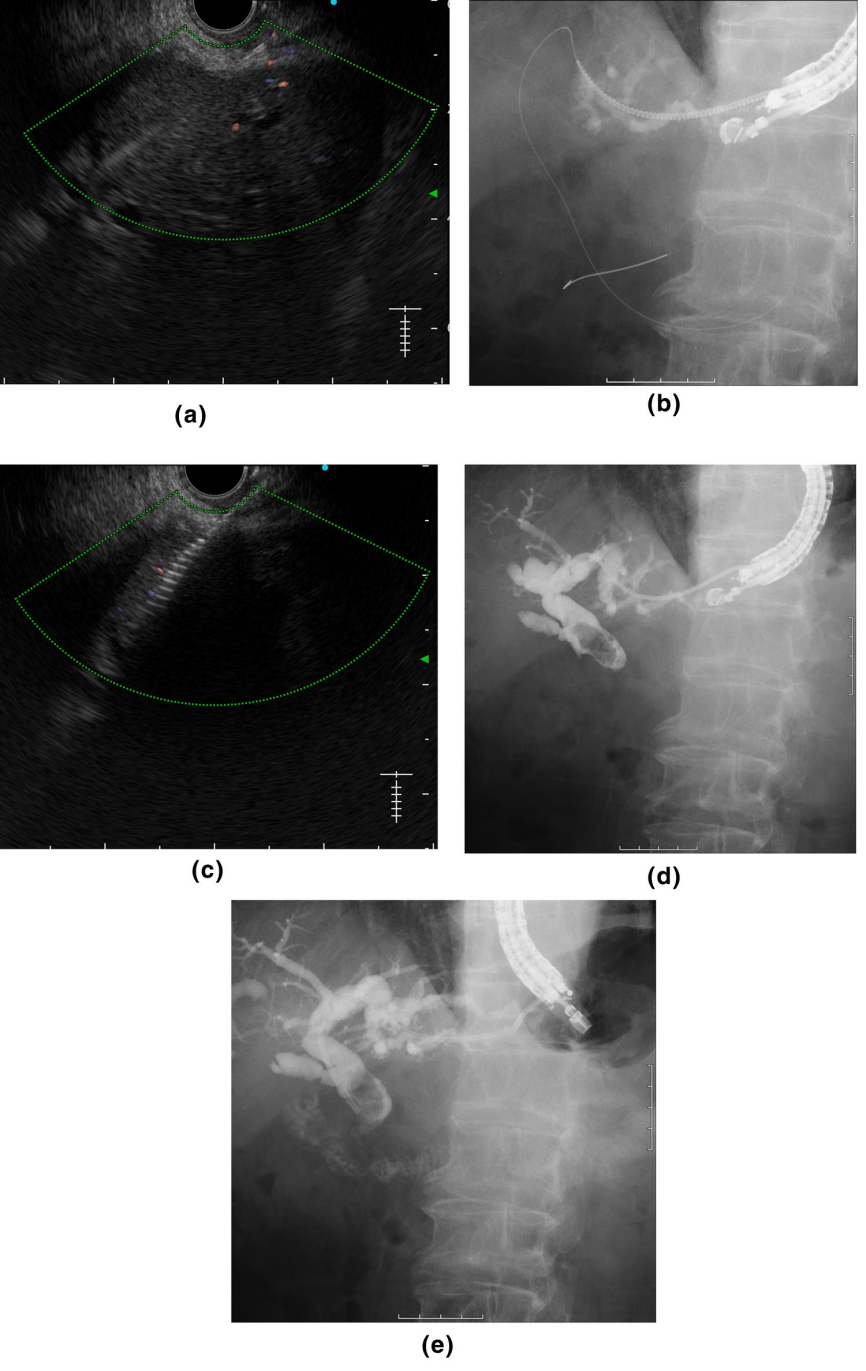
A case of bile duct stones with a surgically altered anatomy (Case No.8 in Table 1). (a) Puncture to the B3 bile duct with a 22‐G needle. (b, c) After inserting a 0.018‐inch guidewire, the punctured tract was dilated using a Tornus ES by turning the handle clockwise. (d) Using cholangiography, common bile duct stones were observed. (e) A plastic stent was placed in the puncture tract

The Tornus ES (Olympus Co., Tokyo, Japan) is a newly developed dilator with a screw‐shaped tapered tip, leading 7 Fr of maximal outer diameter, and two lines up the internal diameter tailored to accommodate 0.025‐ and 0.018‐inch guidewires^15^ (Figure 1). The tip of the dilator is advanced by rotating the proximal handle of the dilator clockwise, and in turn, the puncture tract is dilated^15^ (Figure 2). Push motions along the tract axis are scarcely required, unlike other traditional dilators, since the tip automatically advances due to the screw structure.^15^ After sufficient advancement, the dilator is withdrawn when the assistant rotates it counterclockwise.^15^

### Procedure of EUS‐guided drainage

All procedures for each patient were performed by two doctors among twelve pancreatobiliary endoscopists in our center as an operator and an assistant. Four (Takahisa Ogawa, Yoshihide Kanno, Shinsuke Koshita, and Kei Ito) of the endoscopists were experts who had experienced ≥30 EUS‐guided drainage procedures, and the remaining eight (Hiroaki Kusunose, Toshitaka Sakai, Keisuke Yonamine, Kazuaki Miyamoto, Fumisato Kozakai, Haruka Okano, Hideyuki Anan, and Kento Hosokawa) were trainees who had experienced less than 10 EUS‐guided drainage cases as an operator. Although both experts and trainees were eligible as the first operator in this study, trainees were supervised by an expert and switched when needed.

Puncture of the drainage target was performed using a 19‐G (EZ Shot 3 Plus; Olympus Co.) or 22‐G needle (Expect; Boston Scientific Japan K.K., Tokyo, Japan) under EUS guidance using a convex‐arrayed echoendoscope (GF‐UCT260; Olympus Co.). After confirmation of the drainage target by injecting a contrast medium, a guidewire suitable for the caliber of the needle, i.e., a 0.025‐inch guidewire (VisiGlide2; Olympus Co.) for 19‐G and a 0.018‐inch guidewire (Fielder18; Olympus Co.) for 22‐G, was inserted into the target cavity. Then the puncture tract was dilated using a spiral dilator. A fully covered self‐expandable metal stent or a plastic stent was placed in the puncture tract after dilation. The use of other dilation devices, such as bougie, electrocautery, and balloon dilators, was allowed if dilation was unsuccessful or insufficient using the spiral dilator. The puncture needle and stent were chosen at the discretion of the operators. The decision to perform additional stenting, such as antegrade stenting, depended on the operator as well.

### Outcome measurements

The primary outcome of this study was the technical success rate, which was defined as successful stent placement in the puncture tract. Secondary outcomes were the success rate of dilation using a spiral dilator, procedure time, and adverse events related to the procedure. The procedure time was determined to be the time from the puncture of the drainage target to the removal of the echoendoscope. Adverse events were assessed on the basis of the consensus criteria.^18^

## RESULTS

Details of the patients’ characteristics and clinical outcomes are shown in Table 1. A total of 10 patients (mean age 77 years, six males and four females) were enrolled in this study. Seven patients underwent EUS‐BD (EUS‐HGS for six and EUS‐HJS for one). For all EUS‐BD cases, a 7‐Fr single pigtail PS (Through & Pass, TYPE‐IT; Gadelius Medical Co. Ltd, Tokyo, Japan) was placed in the puncture tract. Among them, an uncovered SEMS (ZEO STENT V; Zeon Medical Inc., Tokyo, Japan) was simultaneously deployed for the distal bile duct stricture in an antegrade manner in 2 EUS‐HGS cases. The etiologies causing the biliary obstruction were pancreatic cancer for three, bile duct cancer for two, gallbladder cancer for one, and bile duct stones for one. The remaining three patients underwent EUS‐GBD for acute cholecystitis complicated with unresectable cancer. For all EUS‐GBD cases, a fully covered SEMS (Covered BileRush Advance; Piolax Medical Devices, Yokohama, Japan) was placed at the puncture tract.

**TABLE 1:** Characteristics and clinical outcomes of the patients

**Case**	**Age**	**Sex**	**Etiology**	**Procedure**	**Operator**	**Assistant**
1	68	F	Pancreatic cancer	EUS‐HGS	Trainee	Expert
2	63	M	Bile duct cancer	EUS‐HGS	Trainee	Expert
3	79	M	Acute cholecystitis	EUS‐GBD	Trainee→Expert	Expert→Trainee
4	74	M	Gallbladder cancer	EUS‐HJS	Trainee	Expert
5	84	F	Pancreatic cancer	EUS‐HGS	Trainee	Expert
6	80	M	Acute cholecystitis	EUS‐GBD	Trainee	Expert
7	73	M	Acute cholecystitis	EUS‐GBD	Trainee	Expert
8	82	M	Bile duct stones	EUS‐HGS	Expert	Trainee
9	76	F	Pancreatic cancer	EUS‐HGS	Trainee	Expert
10	89	F	Bile duct cancer	EUS‐HGS	Trainee	Expert

**Case**	**Puncture needle**	**Stent placement in the puncture tract**	**Type of stent placed in the puncture tract**	**Dilation of the puncture tract using Tornus ES**	**Procedure time**	**Adverse events**	**Additional drainage**
1	19G	Succeeded	7Fr PS	Succeeded	35 min	None	Antegrade stenting of UCSEMS
2	22G	Succeeded	7Fr PS	Succeeded	27 min	None	None
3	19G	Succeeded	10 mm FCSEMS	Succeeded	43 min	None	None
4	19G	Succeeded	7Fr PS	Succeeded	37 min	None	None
5	19G	Succeeded	7Fr PS	Succeeded	25 min	None	Antegrade stenting of UCSEMS
6	22G	Succeeded	10 mm FCSEMS	Succeeded	13 min	None	None
7	19G	Succeeded	8 mm FCSEMS	Succeeded	30 min	None	None
8	22G	Succeeded	7Fr PS	Succeeded	23 min	None	None
9	19G	Succeeded	7Fr PS	Succeeded	15 min	None	None
10	19G	Succeeded	7Fr PS	Succeeded	17 min	None	None

Abbreviations: EUS‐GBD, endoscopic ultrasound‐guided gallbladder drainage; EUS‐HGS, endoscopic ultrasound‐guided hepaticogastrostomy; EUS‐HJS, endoscopic ultrasound‐guided hepaticojejunostomy; F, female; FCSEMS, fully covered self‐expandable metallic stent; Fr, French; G, gauge; M, male; PS, plastic stent; UCSEMS, uncovered self‐expandable metal stent.

The technical success rate and the success rate of dilation using a spiral dilator were both 100%. Among the 10 patients included in this study, for nine patients, the starting operator was a trainee. Of these, for eight patients, the procedure could be completed successfully without switching the operator to an expert. For the remaining patient who underwent EUS‐GBD, the operator needed to be switched from a trainee to an expert due to unsuccessful dilation of the puncture tract. After switching the operator, the procedures, including dilation with a spiral dilator, were successful.

The mean procedure time was 27 min. No adverse events related to the procedure, including peritonitis and bleeding, occurred.

## DISCUSSION

EUS‐guided drainage, including EUS‐BD, EUS‐GBD, EUS‐PD, and EUS‐PCD, has been performed more frequently over the last decade.^1^, ^2^, ^3^, ^4^, ^5^, ^6^, ^7^, ^8^, ^9^, ^10^ Although dedicated devices for EUS‐guided drainage have been developed,^11^, ^12^, ^13^, ^14^, ^15^, ^16^, ^19^ remains a challenging technique, especially for trainee endoscopists.^20^ One of the most difficult steps in EUS‐guided drainage procedures is the dilation of the puncture tract.^12^, ^13^ Existing dilators can be classified into three types, that is, bougie,^12^, ^13^ balloon,^11^ and electrocautery dilators.^14^ Although bougie dilators are the most gentle for surrounding tissues, they need to be most pushed along the tract axis against the resistance. If high resistance impedes smooth advancement, the distance between the dilator's tip and the scope will increase often with the formation of free space in the peritoneal cavity, resulting in low scope stability. Although balloon dilators require a little less power to push, they can still induce a similar situation when the resistance is high. The newly emerged peritoneal space can cause a collection of leaked fluid, and the lack of scope stability can cause procedural failure. Although electrocautery dilators have relatively strong penetrating capabilities, hemorrhage due to a burning effect on the surrounding structures that are not visualized in the EUS image is a concern.^13^ Indeed, Honjo et al.^13^ reported in a retrospective study that electrocautery dilators cause bleeding more often than bougie dilators do.

The spiral dilator has a screw shape, which enables dilation of the puncture tract with simple rotation without needing to push because the tip barely interacts with the tissue. Since the tip advances without needing much power to push it along the tract axis, the scope stability is maintained. In this study, for nine of the 10 enrolled patients, the starting operator was a trainee who had performed less than 10 EUS‐guided drainage cases. Nevertheless, the technical success rate and the success rate for dilation using a spiral dilator were both 100%. Vila et al.^20^ have reported a technical success rate by non‐expert operators of 64.7%. The spiral dilator might have the potential to make procedures easier.

It was only necessary to change the operator from a trainee to an expert endoscopist in one EUS‐GBD case due to unsuccessful dilation of the puncture tract. After switching the operators, the procedures, including dilation of the puncture tract using the spiral dilator, could be completed without much difficulty. For dilation using the spiral dilator, the tip first needs to bite the tissue as screws do. In this case, the trainee endoscopist could not fit the direction of the dilator tip with the puncture tract axis due to insufficient proficiency.

Although stent deployment in the puncture tract becomes easier after sufficient dilation, excessive dilation can cause peritonitis due to leakage of bile or pancreatic juice. In addition, unfavorable injury from the dilation might induce bleeding due to damage to the blood vessels. In this study, no adverse events, including peritonitis and bleeding, were observed. Since a spiral dilator, which is not an electrocautery device, does not cause burning, it might not be likely to trigger bleeding similar to bougie dilators.

It has recently been reported that EUS‐guided drainage is feasible for tiny targets using a small‐caliber needle, that is, a 22‐G needle, combined with a 0.018‐inch guidewire.^19^ When using a 0.018‐inch guidewire, dilators dedicated to this diameter are preferable because the difference between the inner diameter of the dilator and the outer diameter of the guidewire can disrupt the dilation. In our study, three patients underwent EUS‐guided drainage using a 22‐G needle and a 0.018‐inch guidewire. For these patients, a dedicated spiral dilator for a 0.018‐inch guidewire was used, and dilation of the puncture tract was successful.

Our study has several limitations. First, this was a feasibility study with a small number of patients. Although no adverse events, such as peritonitis and bleeding, were observed in this study, they potentially occur in a setting with a larger population. Second, EUS‐CDS, EUS‐PD, and EUS‐PCD cases were not included in this study. Tract dilation through hard pancreatic parenchyma tends to be difficult, making EUS‐PD less feasible. Hara et al.^16^ have reported a successful case of EUS‐PD using a spiral dilator. Although a spiral dilator is thought to be helpful for EUS‐PD, further studies are required. Third, since this was a single‐arm study, a comparison with other dilators, such as bougie, balloon, and electrocautery dilators, was not available.

In conclusion, puncture tract dilation using the spiral dilator was feasible without any adverse events in the limited study cohort. Further studies are required to establish the usefulness of this new device.

## CONFLICT OF INTEREST

Kei Ito has received consultancy fees from ASAHI INTECC., LTD. The other authors declare no conflict of interest.

## FUNDING INFORMATION

None.
